# The Regensburg Dental Trauma Registry: Methodical Framework for the Systematic Collection of Dentoalveolar Trauma Data

**DOI:** 10.3390/jcm13237196

**Published:** 2024-11-27

**Authors:** Matthias Widbiller, Gunnar Huppertz, Karolina Müller, Michael Koller, Torsten E. Reichert, Wolfgang Buchalla, Martyna Smeda

**Affiliations:** 1 Department of Conservative Dentistry and Periodontology, University Hospital Regensburg, Franz-Josef-Strauß-Allee 11, D-93053 Regensburg, Germanymartyna.smeda@ukr.de (M.S.); 2 Center for Clinical Studies, University Hospital Regensburg, Franz-Josef-Strauß-Allee 11, D-93053 Regensburg, Germanymichael.koller@ukr.de (M.K.); 3 Department of Cranio-Maxillofacial Surgery, University Hospital Regensburg, Franz-Josef-Strauß-Allee 11, D-93053 Regensburg, Germany

**Keywords:** tooth injuries, traumatology, registries, patient care, risk factors, prevalence

## Abstract

**Objectives:** Traumatic dental injuries (TDIs) are common, particularly in children and adolescents, and require timely, well-documented treatment for optimal long-term functional and esthetic outcomes. Despite their prevalence, comprehensive data on TDI remain limited. The Regensburg Dental Trauma Registry (RDTR) was established to enable structured data collection, documentation and analysis of dentoalveolar trauma cases to improve both research and clinical practice. **Methods:** The RDTR was developed at the Centre for Dental Traumatology at the University Hospital Regensburg as part of a multi-stage implementation process, which involved creating clinical infrastructure, establishing treatment protocols, providing continuous clinician training, and designing a standardized documentation form to capture essential data, including patient demographics, accident details, clinical assessments, and initial treatment. Data are transferred into a REDCap electronic case report form (eCRF), which is hosted on secure university servers, ensuring efficient administration, controlled access and high data integrity. Quality assurance measures, including automated and manual data checks and regular treatment protocol updates, maintain high data accuracy and consistency. **Results:** This initial methodological report outlines the systematic approach of the RDTR and its potential to generate large datasets. These will enable in-depth analyses of injury patterns, treatment effectiveness, risk factors, and more. Future expansion includes collaboration with additional university hospitals to broaden the dataset and support multi-center approaches. **Conclusions:** The RDTR offers a framework for consistent data collection and quality control, laying the foundation for comprehensive analyses that contribute to the development of preventive strategies and treatment protocols.

## 1. Introduction

The unexpected loss of healthy teeth or tooth fragments as a result of dental trauma is usually a major challenge for the dental practitioner and the patient. Immediate and permanent esthetic and functional restoration is critical to patient satisfaction, self-esteem and quality of life [1,2]. Initial treatment depends on the type and extent of injury and may include restorative, endodontic and surgical measures [3].

Traumatic dental injury (TDI) is common and currently affects more than one billion people worldwide [4]. Although the prevalence of dental trauma in primary (22.7%; 95% CI [confidence interval]: 17.3–28.7%) and permanent teeth (15.2%; 95% CI: 13.0–17.4%) varies in different epidemiological studies, it can be concluded that children and adolescents are particularly affected [4,5,6]. In addition, boys are more likely to be injured than girls, and falls are a common cause of dental injury [7,8,9].

To facilitate the diagnosis and treatment of TDI, numerous classifications have been developed in recent years and updated guidelines have been published by the International Association of Dental Traumatology (IADT) [10,11,12,13]. Overall, these guidelines distinguish between two main types of dental injury, fractures and luxations. Fractures can involve both the crown and the root of a tooth. If both structures are involved, the injury is classified as a crown–root fracture. The extent of damage to the dental tissues is also important and can range from minor injuries to the enamel to complicated fractures involving dentine, cementum and pulp. In contrast, luxation injuries are usually characterized by increased tooth mobility or altered tooth position. However, a concussion or subluxation does not change the position of the tooth, but, at most, increases its mobility. In extrusive, intrusive or lateral luxations, the tooth is displaced. In the case of an avulsion, the tooth is even completely detached from the alveolar socket and is often no longer in the oral cavity [10,11,12,13].

Many studies have been published on the subject of dental trauma over the past decades [14,15,16,17,18,19,20], but the diagnostic assessments, therapeutic options, dental materials and even the etiology of TDI have changed. Qualitative clinical studies focus primarily on specific research questions and are therefore often limited to a specific type of injury or a cohort with a limited number of cases [21,22,23]. Systematic data collection in a study registry, on the other hand, uses a large and continuously growing sample size, providing generalizable findings and allowing for subgroup analyses. Not only can longitudinal data be collected in the context of specific research questions, but also general trends in various aspects of TDI can be followed over time, allowing interesting time-to-event analyses. In general, a study registry provides the opportunity to generate new hypotheses for further investigation [24,25,26,27].

Despite the global paucity of established dental trauma databases [28,29], this issue is particularly pronounced in German-speaking countries, where most studies are single-center, focus on a limited patient cohort, and are conducted within a predefined timeframe [7,30,31]. In response to the need for reliable outcome predictors, improved patient care and new treatment strategies, the Regensburg Dental Trauma Registry (RDTR) was established at the University Hospital of Regensburg. Its aim is to facilitate structured data collection, documentation and analysis of dentoalveolar trauma cases, ultimately improving both research and clinical practice.

## 2. Materials and Methods

### 2.1. Catchment Area and Institutional Background

**Figure 1:**
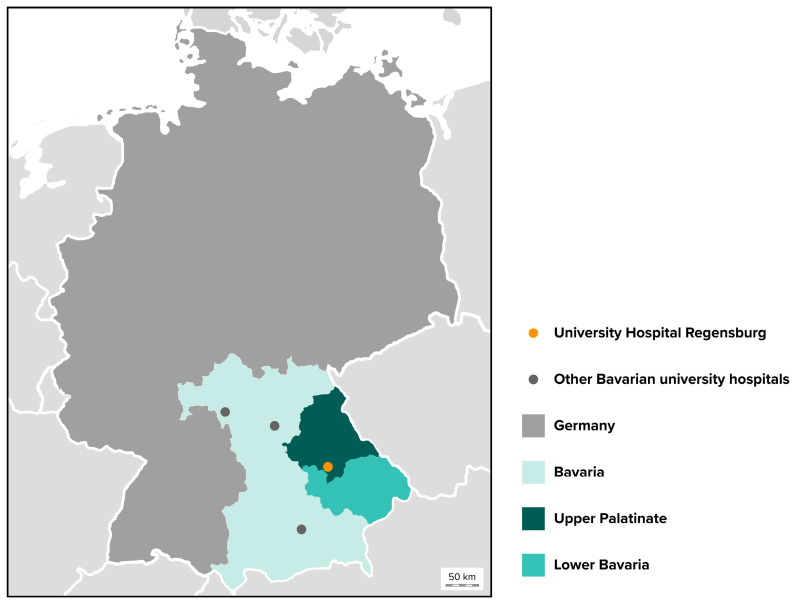
Map of Germany highlighting Bavaria in the southeast. The administrative districts of Upper Palatinate and Lower Bavaria are located in Eastern Bavaria, bordering Austria and the Czech Republic. The University Hospital of Regensburg, one of four Bavarian university hospitals offering dental services, serves as a primary contact for dentoalveolar injuries in Eastern Bavaria.

The Center for Dental Traumatology is a specialized facility at the University Hospital Regensburg, one of four Bavarian university hospitals providing dental services and the central health care provider with the highest level of care in Eastern Bavaria (Figure 1). This includes two Bavarian administrative districts, Upper Palatinate and Lower Bavaria, and covers an area of approximately 20,000 square kilometers. The total population is estimated at 2.3 million [32]. The center is an interdisciplinary collaboration between the Department of Conservative Dentistry and Periodontology and the Department of Oral and Maxillofacial Surgery. Its close integration into the university’s medical complex makes it possible to involve other specialist departments, such as the Department of Orthodontics, the Department of Prosthodontics, the Department of Trauma Surgery or the Department of Pediatrics and Adolescent Medicine, depending on the type and severity of the injury presented. The 24/7 dental as well as maxillofacial service makes the University Hospital an important provider of dental trauma care, not only during working hours, but especially at night and on weekends and holidays. In addition to emergency care, the Center for Dental Traumatology also offers weekly consultation hours for follow-up and treatment planning, and is always available to external colleagues for consultation and advice.

### 2.2. Implementation of the Study Registry

**Figure 2:**
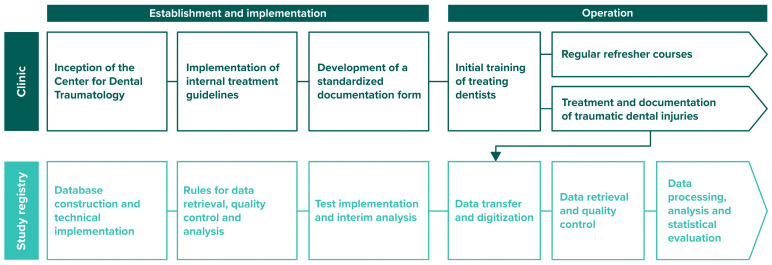
Flowchart outlining the establishment and implementation, and operation of a center for dental traumatology and its study registry.

Clinical patient care and the study registry were established in parallel, with closely coordinated steps. This establishment and implementation phase was followed by the operational phase, during which patient care and data collection continued (Figure 2).

Initially, the clinical focus was on setting up infrastructure and protocols, starting with the establishment of the Centre for Dental Traumatology to provide a dedicated facility for dental trauma care. Next came the implementation of internal treatment guidelines, which standardized procedures for consistent care. Finally, the development of a standardized documentation form allowed for consistent data collection. In parallel, the study registry was set up, which included database construction and technical implementation to manage patient data, establishing rules for data retrieval, quality control and analysis and conducting a test run and interim analysis to validate the system before full operation.

Once implemented, the operational phase includes both clinical and data management activities. Initial training of treating dentists ensures that all staff are familiar with the guidelines, and regular refresher courses maintain high standards in the treatment and documentation of traumatic dental injuries. For data management, data transfer and digitization facilitate efficient record keeping, with routine data retrieval and quality control to ensure accuracy. Finally, data processing, analysis and statistical evaluation allow a comprehensive review of the information collected.

### 2.3. Patient Flow

Patients who have suffered a traumatic dental injury are admitted to the University Hospital in a variety of ways. During normal working hours, they are registered at the desks of the Department of Conservative Dentistry and Periodontology or the Department of Oral and Maxillofacial Surgery. At night, at weekends and on public holidays, the admission takes place through the dental emergency service. In both cases, personal data, insurance details and general medical information are recorded. However, patients with a TDI are not always admitted from the dental clinic. In the case of serious, life-threatening injuries or multiple injuries beyond teeth and oral tissues, emergency general medical care is the first priority. Initial treatment is then provided by the central emergency department of the University Hospital Regensburg. After admission to the hospital by one of the medical departments, specialists from the Center for Dental Traumatology are called in to assess and treat dental injuries.

The registration of all outpatient cases with TDI is carried out continuously by administrative staff at the relevant checkpoints. In order to ensure the registration of inpatients in particular, relevant cases are additionally identified through the billing documentation using the following ICD-10 codes: K08.1 (Loss of teeth due to accident, extraction or local periodontal disease), K08.88 (Other specified disorders of teeth and supporting structures), S02.5 (Fracture of tooth), and S02.6 (Fracture of the mandible).

### 2.4. Standardized Treatment

Traumatized permanent teeth are treated according to the latest version of the national S2 guidelines [33]. Traumatized primary teeth are treated according to the recommendations of the IADT [11]. Internal standard operating procedures are available to each treating dentist to ensure standardized acute care. Regular training and refresher courses ensure a high quality of treatment.

### 2.5. Standardized Data Acquisition

The basis for structured and consistent data collection is a standardized documentation form that was specially developed for this purpose at the Center for Dental Traumatology in 2016 (Supplementary Figure S1). This form has been recommended for the documentation of TDI by the German Society of Endodontology and Dental Traumatology (DGET) and the German Society of Dentistry and Oral Medicine (DGZMK) since 2021. It is also recommended by the national S2-guidelines on dental trauma in permanent teeth to all dentists in hospitals and private practices [33].

First, patients are interviewed about general details of the TDI, and the exact time, place and course of the accident are documented. In addition, the accident is classified as a leisure, work or commuting accident. This is followed by questions about the patient’s general condition, e.g., to exclude concussion and to confirm tetanus vaccination. A detailed intraoral and extraoral clinical report is also obtained, which includes the extent of injury to the teeth, surrounding soft tissues and bone. Injured teeth are assessed for sensitivity, percussion, pocket depth and tooth mobility and categorized as tooth fracture and/or luxation according to the established classification. In the case of tooth avulsion, the extraoral time and storage medium are documented. Radiographic findings, if available, are also recorded. Finally, the initial treatment steps are reported, and further treatment is suggested.

### 2.6. Database Design and Technical Implementation

The study registry is implemented with the assistance of the Center for Clinical Studies at the University Hospital Regensburg, which is responsible for data management and analysis. A study registry database is designed and implemented to enable the systematic collection of trauma data. Records from the standardized documentation form are entered into a web-based electronic case report form (eCRF) using REDCap, a state-of-the-art clinical database management system [34,35]. Study registry data are stored on a server at the University of Regensburg. Access to the data entry interface and the data management system is controlled by the definition and assignment of user roles and user accounts.

Study data are collected by entering numeric and categorical data only, and free text entry is avoided. The full dataset includes rudimentary demographic data (such as month and year of birth, gender, zip code, and insurance status), accident information (such as date and time of accident, location of accident, type and mechanism of accident), information on initial dental trauma care and data on the diagnosis and treatment of affected teeth.

All records are pseudonymized and do not allow any conclusions about identity of the patient. Only authorized study staff can access and manage the study registry records.

### 2.7. Data Handling and Quality Control

To ensure overall data quality, manual and automated data quality controls are implemented and regularly applied to the dataset to ensure maximum completeness and plausibility. After a final and comprehensive data cleaning step, the study registry data are exported into a csv formatted file for further statistical analysis.

### 2.8. Data Processing and Statistical Analysis

The primary aim of the TDI database is to increase knowledge of the prevention and intervention of acute dental trauma. All statistical analyses will be performed using the SPSS software package (version 28 or higher). Syntaxes will be developed to ensure high quality and transparency in data processing and standardized data analysis. Descriptive analyses will be performed at the patient and tooth level for demographic and clinical characteristics using frequency (n), percentage (%), mean (M), standard deviation (SD), median (MED), interquartile range (IQR) and range (min/max) depending on the scale level and distribution of the parameters. The statistical analysis plan will be developed according to the pending research questions.

## 3. Discussion

Since 2016, the Regensburg Dental Trauma Registry has systematically documented over 2000 traumatic dental injuries (as of November 2024), and a growing number of dental trauma cases are continuously being treated and recorded at the Centre for Dental Traumatology at the Regensburg University Hospital, all of which contribute to a growing study register. Based on current performance, we expect to be able to include at least 60 new cases in the registry each quarter. This constantly evolving database forms an important basis for future scientific investigations and research efforts.

However, a database is only as reliable as the quality of the information it contains. In the case of the RDTR, these data come from standardized documentation forms, typically completed by the dentist during the initial treatment following a dental accident. Therefore, accurate and thorough form completion is important and has been identified as a key challenge of the project to date. Correct entry by the treating dentist is crucial, as both incomplete patient histories or insufficient training in dental traumatology can compromise the quality of information provided in the forms. Thus, one lesson learnt was the necessity of comprehensive onboarding and intensive training to maintain high treatment quality and precise data documentation, especially in large dental clinics with frequent staff turnover. In this context, we provide detailed background of the registry and information on the specific documentation required to make the transition to the eCRF as easy and seamless as possible. Another major challenge impacting data quality is missing patient information, often due to memory lapses regarding specifics like the time and circumstances of the accident, leading to data gaps. Nonetheless, errors in form completion have been rare, thanks to systematic quality control measures conducted during data transfer to the REDCap database management system. These checks are designed to prevent inconsistencies and ensure data integrity and reliability, such as identifying logical discrepancies like mismatched treatment and accident times or recording permanent teeth involvement in toddlers. Consequently, the RDTR upholds high data quality, which is essential for subsequent analysis and research [34,35].

The RDTR allows analysis of key demographic data, causes of dental accidents and prevalence of injury patterns. This information is essential to assess risk factors for TDI and to mitigate dentoalveolar trauma. One of the main objectives is to use the evidence from the registry to develop and implement effective prevention and treatment strategies that will improve patient care, particularly for children and adolescents, both in Eastern Bavaria and beyond. In addition, more specific questions will be explored, such as changes in the nature of dental injuries, e.g., in terms of injury patterns, age groups affected and accident locations, during the COVID-19 lockdown periods affecting German society in 2020 and 2021.

In addition to cross-sectional data, the project aims to track changes in dental injury patterns over time, influenced by factors such as innovations like e-scooters and new sports trends, or even to reflect wider changes in areas such as health care or public safety. Specific research questions will also be explored to provide deeper insights into the long-term outcomes of certain dental injuries and their impact on patients’ quality of life. This includes a focus not only on tooth survival but also on complications such as pulp loss, pulp canal obliteration, and pathological resorption, advancing the field of dental traumatology [36].

Future initiatives include the targeted collection of longitudinal data from routine or research-initiated follow-up visits. The flexibility of the system also allows for the integration of multiple centers, creating new research opportunities. In particular, preparations are underway to include all four university hospitals providing dental services in Bavaria, enabling supra-regional data collection across southeast Germany. This expansion is expected to increase the scope of research and provide a more comprehensive understanding of dental trauma on a larger scale, minimizing potential regional or clinic-specific effects.

## 4. Conclusions

In summary, the Regensburg Dental Trauma Registry offers significant scientific, economic, institutional and social benefits. Its primary goal is to advance knowledge in dental traumatology by addressing specific research questions. Furthermore, it aims to identify gaps in health care, improve prevention efforts, and increase awareness of acute dental trauma interventions among patients and health care professionals.

## Data Availability

The datasets presented in this article are not readily available because the data are part of an ongoing study.

## Supplementary Materials

The following supporting information can be downloaded at: https://www.mdpi.com/article/10.3390/jcm13237196/s1. Supplementary Figure S1: Standardized documentation form.
