# Supplementary material for: The Regensburg Dental Trauma Registry: Methodical Framework for the Systematic Collection of Dentoalveolar Trauma Data
Source: J Clin Med. 2024 Nov 27;13(23):7196. doi: 10.3390/jcm13237196 (PMC11642096; doi:10.3390/jcm13237196)
Supplement: Supplementary file 1 [file jcm-13-07196-s001.zip › jcm-3315085-supplementary.pdf]

Patientendaten

Name: ______________________

Vorname: ______________________

Geburtsdatum: ______________________

Krankenkasse: ______________________

Befundbogen Zahntrauma

Deutsche Gesellschaft für Endodontologie und Zahnärztliche Traumatologie
Erstellung: Prof. Dr. M. Widbiller in Kooperation mit den Universitäten/
Zahnunfallzentren Regensburg, Würzburg, Erlangen, Basel

Datum: ______________ Uhrzeit: ______________ Behandler: ______________

Unfalldokumentation ☐ Freizeitunfall ☐ Arbeits-, Schul- oder Wegeunfall (etc.)

Datum: ______________ Uhrzeit: ______________

Ort bzw. Weg: ______________________

Hergang: ______________________

____

Unfallanamnese

☐ Bewusstlosigkeit ☐ retrograde Amnesie ☐ Foetor alcoholicus

☐ Übelkeit/Erbrechen ☐ kein Tetanusschutz vorhanden

Traumabefund

	15/55	14/54	13/53	12/52	11/51	21/61	22/62	23/63	24/64	25/65
Dislokationsverletzung [mm]*										
Sensibilität										
Perkussion										
Pulpaexposition										
Lockerung (0 bis III)										
Sondierungstiefe [mm]										

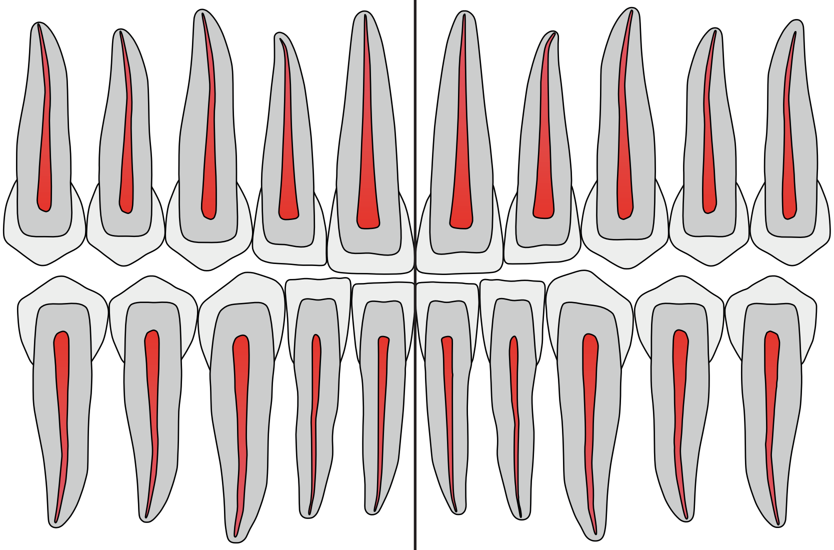

	45/85	44/84	43/83	42/82	41/81	31/71	32/72	33/73	34/74	35/75
Dislokationsverletzung [mm]*										
Sensibilität										
Perkussion										
Pulpaexposition										
Lockerung (0 bis III)										
Sondierungstiefe [mm]										

* Bei Zahnfrakturen durchgängige Verlaufslinien einzeichnen, bei Infrakturen gestrichelte. Bei Dislokationsverletzungen das Abkürzungszeichen (s.u.), die Richtung (m, d, v, o) sowie das Ausmaß der Dislokation in [mm] in die vorgesehne Spalte eintragen (z.B. I/2 oder D/o/3).

K für Konkussion **L** für Lockerung **D** für Dislokation **E** für Extrusion **I** für Intrusion **A** für Avulsion

Avulsionsanamnese

Trockenlagerungsdauer: ______________________

Transportmedien (Lagerungsdauer): ______________________

Röntgenbefund ☐ Zahnfilm (__________) ☐ Orthopantomogramm

____

____

____

Weitere Befunde

Alveolarknochen: ______________________

____

____

Kiefergelenk: ______________________

____

____

Alveolarmukosa: ______________________

____

____

Lippen: ______________________

____

____

Gesichtshaut: ______________________

____

Initialtherapie

____

____

____

Weiterbehandlung

____

____

____

____

Formular zurücksetzen | Datei Drucken | Speichern unter
